# Guided Wave Ultrasonic Testing for Crack Detection in Polyethylene Pipes: Laboratory Experiments and Numerical Modeling

**DOI:** 10.3390/s23115131

**Published:** 2023-05-27

**Authors:** Jay Shah, Said El-Hawwat, Hao Wang

**Affiliations:** 1 Centre of Advance Infrastructure and Transportation, Rutgers—The State University of New Jersey, Piscataway, NJ 08854, USA; 2 Department of Civil and Environmental Engineering, Rutgers—The State University of New Jersey, Piscataway, NJ 08854, USA

**Keywords:** polyethylene, plastic pipe, ultrasonic testing, finite element model, crack detection

## Abstract

The use of guided wave-based Ultrasonic Testing (UT) for monitoring Polyethylene (PE) pipes is mostly restricted to detecting defects in welded zones, despite its diversified success in monitoring metallic pipes. PE’s viscoelastic behavior and semi-crystalline structure make it prone to crack formation under extreme loads and environmental factors, which is a leading cause of pipeline failure. This state-of-the-art study aims to demonstrate the potential of UT for detecting cracks in non-welded regions of natural gas PE pipes. Laboratory experiments were conducted using a UT system consisting of low-cost piezoceramic transducers assembled in a pitch-catch configuration. The amplitude of the transmitted wave was analyzed to study wave interaction with cracks of different geometries. The frequency of the inspecting signal was optimized through wave dispersion and attenuation analysis, guiding the selection of third- and fourth- order longitudinal modes for the study. The findings revealed that cracks with lengths equal to or greater than the wavelength of the interacting mode were more easily detectable, while smaller crack lengths required greater crack depths for detection. However, there were potential limitations in the proposed technique related to crack orientation. These insights were validated using a finite element-based numerical model, confirming the potential of UT for detecting cracks in PE pipes.

## 1. Introduction

Polyethylene (PE) has been widely utilized in natural gas distribution pipelines due to its cost-effectiveness, flexibility, lightweight nature, and excellent resistance to chemical corrosion [1]. The ethylene monomer units and linear chain structure give PE higher flexibility and impact resistance compared to other plastic pipes such as Polyvinyl Chloride (PVC), known for its rigid nature. When properly maintained, PE pipes can provide a service life of up to 50 years. As the understanding of PE as a material has improved, the development of enhanced PE variants [2] has resulted in approximately 90–95% of new natural gas pipelines being constructed using PE [3]. However, despite its numerous advantages, PE is a viscoelastic material that exhibits high sensitivity to extreme operational loads and environmental factors. Consequently, it is susceptible to developing defects during its service life [4,5]. Additionally, the presence of PE pipelines in densely urbanized areas raises safety concerns due to the potential for gas leaks or pipe bursts from existing defects. Given the flammable nature of such incidents, they pose a significant threat to society and can have substantial economic consequences. Therefore, it is essential to develop efficient strategies for pipeline monitoring to detect defects at early stages and prevent gas pipeline failures.

Surveys have consistently identified external surface defects as a leading cause of PE pipeline failures [6]. Among these defects, external cracks are the most common and can result from various factors, including third-party damage, manufacturing defects, soil differential settlement, and tensile stress induced by internal pressure [7,8,9,10]. Given the semi-crystalline nature of PE, which exhibits time-dependent creep-stress behavior, it is crucial to detect cracks in their early stages due to their significance in the quasi-brittle and brittle failure modes of PE [11,12,13]. The failure mechanisms of PE pipes can be categorized into three stages: ductile, quasi-brittle, and brittle failure. Ductile failure typically occurs under high hoop stress and has a relatively short failure time, often manifesting as bending or kinking at the failure site [14]. As the failure curve progresses, it transitions to the quasi-brittle stage characterized by the initiation of slow crack growth (SCG), which gradually propagates under constant low-stress conditions [15]. This phase occurs over a longer failure time and is generally considered a benchmark for assessing the long-term performance of the pipe. Ultimately, brittle failure occurs in a nearly load-independent manner, primarily resulting from multiple instances of SCG or the effects of chemical and thermal aging on PE [16].

Prior to installation, PE pipes undergo rigorous hydrostatic testing to project their long-term performance using the rate process method and extrapolation technique [17,18]. The hydrostatic test subjects the pipes to elevated internal pressures to assess their strength and integrity under simulated operating conditions, whereas extrapolation techniques are subsequently applied to analyze the hydrostatic strength test data, aiding in the estimation of the pipes’ behavior over extended periods. While hydrostatic tests are useful in assessing the overall strength of the pipe, they do not provide any information on pre-existing cracks, making it necessary to develop fracture mechanics models to study the susceptibility of PE pipes to crack propagation [19,20,21,22]. By quantifying the crack growth rate and estimating the time to failure, these models aid in developing effective maintenance strategies of pipelines [23]. Despite repeated emphasis on the early detection of cracks in the literature, reported studies on crack detection in PE pipes have primarily concentrated on identifying cracks in welded regions [24,25]. Therefore, it is of interest for the research community to expand the existing methodologies for crack detection in non-welded regions of pipes. The findings will contribute to the efficient inspection of natural gas pipelines. 

A wide range of methodologies are available for the condition monitoring of pipes in the literature, each with its unique advantages and limitations. Hence, selecting the appropriate technique is crucial for the successful detection of cracks on the pipe’s body. Visual inspection is typically the first step for any structural evaluation, requiring an inspector visiting the site to isolate the defected section [26]. However, this approach is limited to detecting surface defects provided there are obvious signs of defect, and the outcome is subjective to the inspector’s expertise. Given the extensive network of natural gas pipelines, visual inspection is highly time-inefficient and costly due to its manual nature. The radiography technique is another non-destructive technique that uses X-rays and Gamma rays to identify internal cracks in structures and is mainly employed to detect weld defects, cracks, and the presence of foreign objects in the welded regions of PE pipes. The instruments used in this technique are bulky and non-portable for site inspection while also posing a potential threat of radiation hazard [27,28]. Hence, this technique is also unsuitable for monitoring PE pipes for cracks in field conditions. Infrared (IR) thermography, known for its real-time inspection, relies on the thermal profile of the defected region in PE pipe as the existing defect changes the thermal signature relative to the material in its vicinity [29,30]. However, this approach also requires relatively expensive equipment, and the results are subjective to temperature fluctuations on the test day. In addition, the resolution of the IR camera and its accessibility to the surface can also limit this application. Microwave technology is recognized for its ability to detect defects in a non-contact manner [31,32]. It uses a surface-penetrating radar combined with a microwave probe to record reflectivity patterns from the region under inspection. The defect resolution is sensitive to the probe distance from the surface, and near-field defect detection can be challenging unless the inspection frequency and probe distance are optimized. Given the outcome’s sensitivity to probing distance and precise wave generation, specialized equipment and sensors are needed, hence increasing the equipment cost. In summary, for crack detection in PE pipes in a long-distributed pipeline network, a low cost and time efficient method which can offer long-range pipeline inspection in a non-invasive manner is generally preferred.

Guided wave-based ultrasound testing (UT) is one such established approach for the inspection of metallic pipelines, ranging from the detection of cracks, weld defects, and corrosion [33]. However, the application of UT in monitoring PE pipes is still in its early stages. Intuitively, the reason can be associated with the low density and elastic modulus of PE [34,35], which results in the generation of highly attenuative and dispersive guided wave modes in PE. Guided wave inspection typically initiates with the selection of specimen-specific wave modes with low dispersion and attenuation characteristics, hence facilitating long-range inspection in structures. The wave propagation at the selected modes is then monitored for its interaction with potential defects in specimens with a pitch-catch or pulse-echo approach [36]. To the authors’ best knowledge, only a handful of studies have reported the use of guided wave-based UT for crack monitoring in PE pipes as discussed next, hence making it an area of interest for further research.

Lowe et al. [37] successfully demonstrated the use of low-frequency longitudinal wave modes to monitor PE pipes of up to 700 mm length from a single point of inspection using micro-flexible transducers configured in a ring setup. However, the potential of this methodology was not tested for its defect detection ability in the specimen; another scope for advancement is to include material damping properties which was also not considered in their numerical model. This concept of wave propagation was extended to crack detection in polyvinyl chloride (PVC) pipes [38] using a nonlinear ultrasonic wave modulation technique. The study revealed that the frequency spectrum of a probing signal comprised of two different frequencies was sensitive to its interaction with cracks, resulting in the generation of new harmonics. Another similar study reported the use of non-collinear ultrasound wave mixing approach in a pulse-echo setting to monitor wave parameters (phase velocity, dispersion, and attenuation) that are sensitive to physical aging of PVC pipe [39]. While the use of nonlinear ultrasonic method has been repeatedly reported for its ability to detect cracks in their incipient stages in metallic pipes [40], its implementation on large-diameter PE pipes (more wave attenuation) is yet to be tested.

This state-of-the-art investigation aims to investigate the capabilities of UT for crack detection in PE pipes through a comprehensive approach involving laboratory experiments and numerical modelling. The study focuses on UT-based inspection of a natural-gas-distribution PE pipe with cracks of varying geometries and orientations. A cost-effective ultrasonic setup will be assembled in a pitch-catch configuration to monitor wave propagation, and the transmitted signal’s amplitude will be analyzed to investigate the impact of cracks on wave interaction. The findings will be further validated using a robust numerical model that incorporates material damping properties to accurately represent experimental conditions. The planned investigation will consist of several key phases: (1) The theoretical analysis of wave propagation in PE pipes, (2) selection of optimal inspection wave modes, (3) laboratory experiments, (4) the development of a robust numerical model, (5) the validation of experimental results, and (6) the discussion of the insights gained.

## 2. Theoretical Analysis

### 2.1. Fundamentals of Wave Propagation in Pipes

It is crucial to understand the fundamental theory of elastic wave propagation in pipes to optimize inspection parameters for ultrasonic testing (UT), such as signal frequency, distance between the transmitter and receiver, and signal shape. The propagating stress wave in the pipe follows Navier’s Stokes relation as shown in Equation (1) [37,41].

(1) $$(\lambda +\mu )\nabla \left(\nabla \cdot u^{\prime }\right)+\mu \nabla^{2}u^{\prime }=\rho \frac{\partial^{2}u^{\prime }}{\partial t^{2}}$$

where *λ*, *µ* represent Lamé constants, *u’* is the displacement vector, ∇^2^ denotes Laplace operator, and *ρ* stands for material density. 

The solutions for displacement (*u′*) are further developed by using Helmholtz decomposition as the gradient of compressional scalar potential (ø), and equivoluminal vector potential (*H*) as described in Equation (2). Through the substitution of Helmholtz decomposition potentials into the Navier–Stokes equation, one can represent two potentials as a function of longitudinal and shear wave modes as described in Equations (3)–(6)

(2) $$u^{\prime }=\nabla ø+\nabla \times H,\nabla \cdot H=0$$

(3) $$\nabla^{2}\emptyset =\frac{1}{c_{L}^{2}}\frac{\partial^{2}\emptyset }{{\partial t}^{2}}$$

(4) $$\nabla^{2}H=\frac{1}{c_{T}^{2}}\frac{\partial^{2}H}{{\partial t}^{2}}$$

(5) $$c_{L}^{2}=\frac{\lambda +2\mu }{\rho }$$

(6) $$c_{T}^{2}=\frac{\mu }{\rho }$$

where *c_L_* and *c_T_* denote longitudinal and shear mode velocities.

These two modes can propagate in any direction in an infinite media; however, given the constraints of cylindrical geometry in pipes, these modes are limited to advance in either axisymmetric or non-axisymmetric directions. These boundary constraints result in multiple modes of vibrations, namely, the longitudinal mode L(m,n), torsional mode T(m,n), and flexural modes F(m,n), where m represents the circumferential order and n represents the incremental group order number. Corresponding to the thickness and the inner diameter of the pipe, different wave modes can be excited at a given excitation frequency. Therefore, it is critical to conduct dispersion analysis of different modes for a given specimen to select the optimal wave mode for inspection.

### 2.2. Selection of Inspection Parameters

The specimen chosen in this study is a PE pipe with an internal radius of 80 mm and 20 mm thickness. Such pipes are typically used as gas service pipelines. The excitation frequency, selection of wave modes, and inspection length can be fine-tuned by analyzing the dispersion and attenuation curves of different wave modes. An open-source software, GUIGUW, was used to generate data points for dispersion curves [42]. The properties listed in Table 1 were obtained from a previous study on PE [43] to provide preliminary understanding of wave propagation. 

**Figure 1:**
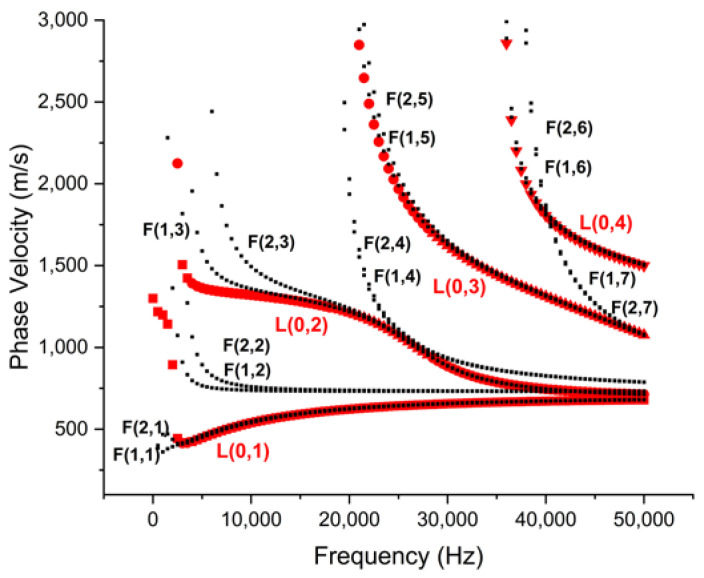
Phase velocity dispersion curves of the pipe specimen.

**Figure 2:**
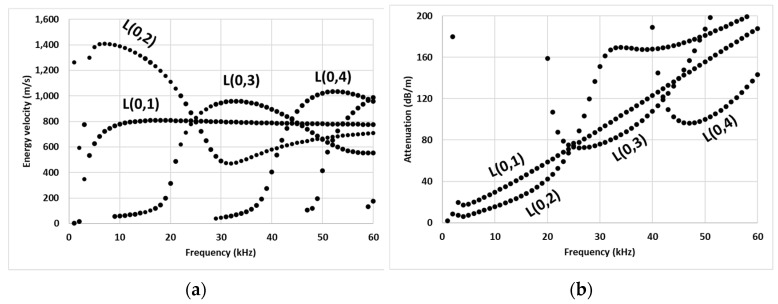
Illustration of (**a**) energy velocity, and (**b**) attenuation curves for PE pipe.

Figure 1 shows the phase velocity dispersion curve for the studied pipe. The complexity of signal analysis using such wave modes can be projected from the co-existence of multiple wave modes with relatively similar phase velocities over a wide range of ultrasonic frequencies. For example, right from the start of the ultrasonic frequency range (20 kHz), the first two longitudinal modes, the L(0,1) and L(0,2) modes, are plagued with the simultaneous arrival of multiple flexural modes. Furthermore, these modes are relatively more dispersive in the ultrasonic range as can be seen in energy velocity curves in Figure 2a. The curves for the L(0,1) and L(0,2) modes intersect with L(0,3) mode at 25 kHz, making it challenging to identify these modes in practice. Practically, the L(0,3) mode at 35 kHz and L(0,4) mode at 50 kHz are the first two relatively less-dispersive modes with distinctive energy velocities than other coexisting modes. In experiments, these modes can be identified as the first envelope in the transmitted signal. The selection of these modes for inspection is also supported by their attenuation characteristics. From attenuation curves in Figure 2b, it is evident that above 20 kHz, the L(0,3) mode is the least attenuative longitudinal mode at 35 kHz with an attenuation of 83 dB/m, whereas L(0,4) mode at 50 kHz is the next least attenuative mode with an attenuation of 95 dB/m. Hence, based on the above discussion, the L(0,3) mode at 35 kHz and L(0,4) mode at 50 kHz were finalized for studying crack-wave interaction in this study.

## 3. Laboratory Experiments of Ultrasonic Tests

### 3.1. Experimental Setup

**Figure 3:**
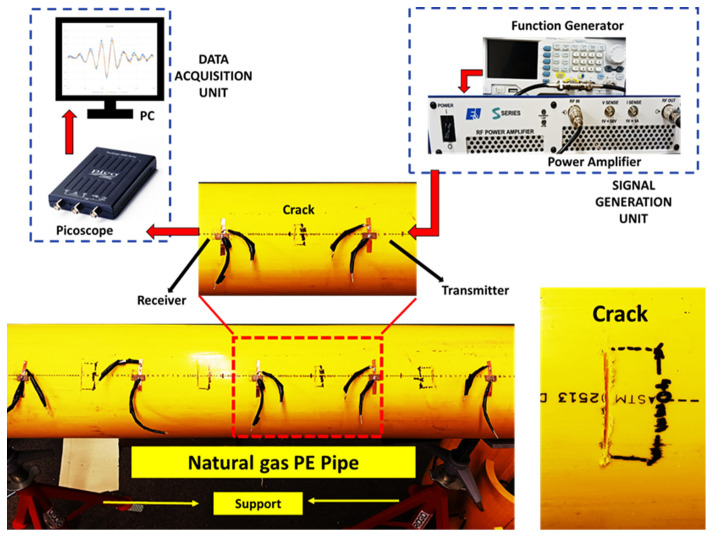
Experimental setup for ultrasonic testing of the PE pipe with crack.

The experimental setup for UT was assembled as shown in Figure 3 where PE pipe was placed on two supports. Piezoceramic patches (shear c255, 18 mm × 9 mm × 1.76 mm by PI Ceramics, Auburn, MA, USA) were used to transmit and record the ultrasonic stress wave signal. These patches have polarization along the 18 mm dimension, making the geometry suitable for capturing longitudinal modes. The patches were attached to the surface of the pipe using a PE-specific adhesive, which was left to cure for 24 h, ensuring its compatibility with the PE material. It is noted that the commonly available adhesives in the market were found to be incompatible with PE material, which might be due to less surface energy of PE being available for adhesion [44]. To mitigate another challenge associated with the heat sensitivity of PE, the connecting cables were soldered to the electrodes of the patches before attaching them to the pipe using the adhesive. 

A function generator (RIGOL DG1035Z, Portland, OR, USA) was used to generate customized signals which were further amplified by a high-voltage power amplifier (E&I 1000S04, Electronics & Innovation, Ltd., Rochester, NY, USA). The amplified signal was fed to the transmitter patch and the propagation of signal was recorded with another identical patch. No amplification was required at the receiver’s end. The recorded signal was digitized for further analysis using an oscilloscope (Picoscope 2204A, Pico Technology, Tyler, TX, USA) and data acquisition was performed with the in-house software named Picoscope, which is available on the vendor’s website, and a computer. The circumferential cracks were fabricated in the middle of the receiver and transmitter and perpendicular to the axis of pipe (longitudinal wave propagation direction). Only perpendicular cracks were considered to test the hypothesis, citing limited reports on the success of UT for crack detection in PE pipes. Cracks with different lengths and depths were fabricated on the external surface of the PE pipe using high-RPM power tools while the crack width was kept constant at 2 mm for all the cases. However, it was found that achieving the repeated accuracy of crack depth was challenging with the handheld tool; hence, a measurement error of ±5% crack depth was considered in the analysis.

### 3.2. Experimental Observations on Effect of Crack Geometry on Wave Propagation

A 5-cycle Hanning window signal with central frequency of 35 kHz and 50 kHz was used to actuate ultrasonic stress wave into the pipe specimen. The signals were amplified with a 60 dB gain and signal propagation was recorded for a 200 mm transmission length. This length was chosen considering the attenuation of wave modes and the significantly high signal amplification requirements in experimental conditions. In post-processing, the recorded signals were passed through a bandpass filter to eliminate unwanted frequency noise, a common phenomenon with high-gain amplifiers. In addition, to eliminate potential instrument sensitivity challenges, traces of 96 measurements for each considered case were averaged to generate a waveform representing a single experimental case.

**Figure 4:**
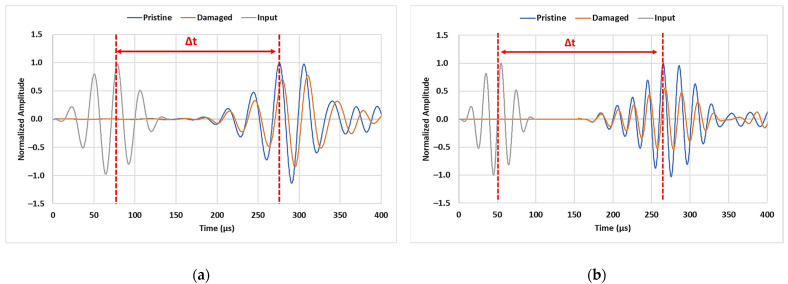
Transmitted signal at (**a**) 35 kHz and (**b**) 50 kHz excitation.

Figure 4a,b shows the transmission of 35 kHz and 50 kHz signal in terms of normalized amplitudes for the PE pipe specimen in its pristine state and one of the damaged conditions (crack length: 50 mm, crack width: 2 mm, crack depth: 8 mm). As anticipated from dispersion analysis, the reception of the first transmitted envelopes with the highest peak at 278 µs and 265 µs marked the arrival of the L(0,3) and L(0,4) modes. The mode’s group velocity was calculated using the time difference (∆t in the figure) between the highest input peak and the amplitude of the peak corresponding to the anticipated arrival time of the mode of interest. These arrival times correspond to the propagation velocity of 970 m/s and 959 m/s which was within ±10% range when compared to the theoretical value of 983 m/s and 1000 m/s obtained from dispersion analysis. The peak-to-peak amplitude of peaks corresponding to the chosen longitudinal mode was used to monitor the wave-crack interaction. The amplitude decay of 42% in the L(0,3) peak and 43% in L(0,4) peak can be observed with a marginal shift in the arrival time of the peak when the crack was introduced in the ultrasonic wave transmission path.

The cracks with different lengths and depths are used to study the effect of crack geometry on wave propagation. CLs of 30 mm, 40 mm, and 50 mm with varying depths of 10%, 20%, 30%, and 40% of the pipe wall thickness were considered in this study. These cover a wide range of CLs relative to the wavelength of inspection modes. The L(0,3) mode at 35 kHz had a wavelength of approximately 48 mm, whereas the L(0,4) mode at 50 kHz had a wavelength of 38 mm which were calculated by using the phase velocity of these modes as shown in Figure 1. The CD was controlled using a high-RPM power-cutting tool.

**Figure 5:**
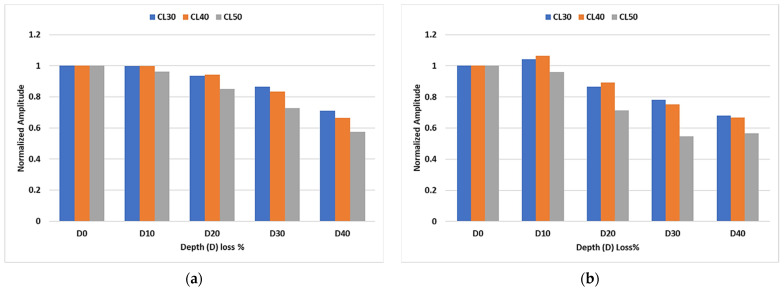
Experimental trend for inspection with (**a**) 35 kHz and (**b**) 50 kHz signal.

Figure 5 shows the trend in the observed peaks of transmitted signals that are normalized to the peak at the pristine state (depth loss 0%). The peak corresponding to the L(0,3) mode at 35 kHz excitation (Figure 5a) shows a progressive amplitude decay trend at a depth loss of 20% of the pipe wall thickness and beyond. This variation is more prominent for the CL of 50 mm at all the considered CDs. On the contrary, this decay trend is relatively sharper for the inspection with a 50 kHz signal (Figure 5b), while another distinct observation is the trend towards the saturation in amplitude decay at a depth loss increasing from 30% to 40% of the pipe wall thickness. Similar to the 35 kHz signal, the variation in the transmitted signal is more prominent for the 50 mm CL. This highlights the fact the CLs close to the wavelength of inspecting mode are easier to detect, whereas a higher CD is required to effectively observe this interaction at a lower CL.

## 4. Development and Validation of Numerical Models

### 4.1. Numerical Modeling

**Figure 6:**
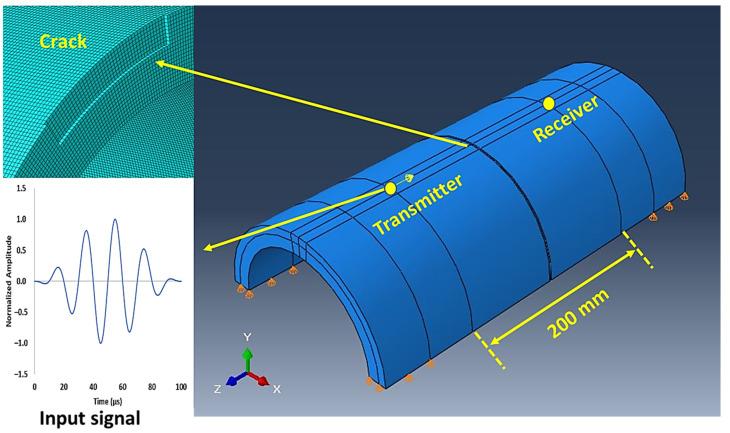
ABAQUS model for the pipe specimen.

The wave propagation in the pipe specimen was numerically simulated using the finite element method, which is known for its capability to model complex structures [45]. The commercial software ABAQUS [46] was employed for this numerical simulation. To avoid a computationally expensive model, only half the section of the pipe was modeled, as shown in Figure 6. Although the wave propagation within 200 mm distance needed to be simulated, extra lengths before the transmitter and after the receiver were included to accommodate the requirements of supports at two ends and to mitigate the possibility of interference from boundary reflections. A boundary condition constraining the vertical displacements was applied on the support ends of the pipe specimen. The cracks were simulated in the model by removing the elements in specific crack geometries. For the modelling of mechanical excitation from piezoelectric transducers, axial point loads in the Hanning window were applied at the transmitter location to excite ultrasonic signals in the specimen. Similarly, axial displacements at the receiver node were recorded to capture the transmitted signal and later exported for the analysis. 

For viscoelastic materials such as PE, where the dissipation of wave energy is relatively higher than in stiffer materials, it was crucial to introduce material damping in the model to capture the experimental trend accurately. However, the damping properties for PE in the frequency range used in this study are not widely reported. To address this issue, previous studies [47,48] were used as a basis for optimizing material damping, where overall attenuation during wave propagation was represented as a function of the geometric spreading of the wavefront with increasing propagation distance, and a viscous damping term defined using the Rayleigh damping model. The corresponding parameters in these studies were fine-tuned through the curve-fitting approach to capture the experimental trend of wave mode amplitude reduction with the propagation distance. Similar to those studies, the attenuation characteristic of the longitudinal mode is represented as a function of geometric spreading of the wavefront and wave dispersion due to frequency-dependent mode velocities, which is approximated through the Rayleigh damping model as shown in Equations (7) and (8),

(7) $$L(m,n)\left\{r,\omega \right\}\propto G\left(r\right)e^{-\eta r}$$

(8) $$\eta =\frac{1}{2c}\left(\alpha +\beta \omega^{2}\right)$$

where L(m,n) is the longitudinal mode, *r* is the propagated distance, *ω* is the angular frequency, *G* represents the geometric spreading of the wavefront, *η* is the damping coefficient of the material, *α* and *β* are Rayleigh damping parameters, and *c* is the wave velocity.

Since the propagation distance is fixed in this study, only the damping coefficient (*η*) was fine-tuned to capture experimental observations. Two simplifications in the selection of Rayleigh parameters were considered to improve the model’s ability to capture experimental observations. First, the shape of the transmitted envelope should agree with experimental observations in the pristine conditions. Second, the ratio of the peaks between the L(0,3) mode at 35 kHz and L(0,4) mode at 50 kHz from experimental observations should match that from numerical models, which eventually can be written as a function of Rayleigh parameters as shown in Equation (9). After testing several combinations of Rayleigh parameters, fixing the *β* value to zero and using *α* value as 15,000 for 35 kHz and 22,000 for 50 kHz excitation satisfied the above two criteria. A relatively similar order of Rayleigh parameters (*α* and *β* values of 120,000 and 0) were reported to capture wave dispersion in reinforced fiber polymer [47].

(9) $$\frac{{L(0,3)}_{exp}}{{L(0,4)}_{exp}}=\frac{{L(0,3)}_{num}\{\alpha ,\beta \}}{{L\left(0,4\right)}_{num}\{\alpha ,\beta \}}$$

where L(m,n)_exp_ and L(m,n)_num_ are peak amplitudes of the receiver signals from the experimental observations and numerical models, respectively.

Another effort to improve the model accuracy and reduce numerical fluctuations was to include a generous number of mesh elements within a given wavelength, as shown in Equations (10) and (11).

(10) $$dl=\frac{\lambda_{mode}}{N}$$

(11) $$\lambda_{mode}=\frac{c_{phase}}{f}$$

where *dl* is mesh element size, *N* is number of mesh elements within a wavelength, *λ_mode_* is the wavelength, and *c_phase_* is the phase velocity.

**Figure 7:**
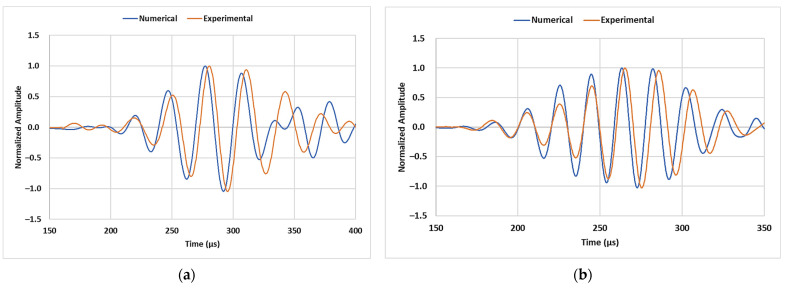
Comparison of normalized amplitudes of transmitted signals at (**a**) 35 kHz and (**b**) 50 kHz from experimental observations and numerical models.

A mesh element size of 1 mm was finalized, which satisfies the requirement of having at least 40 mesh elements (*N*) within the wavelength of the interacting mode (*λ_mode_*) at the inspection frequency (*f*). Standard C3D8R mesh elements were used in the model. Eventually, a maximum time step of 0.1 µs was chosen to obtain stability in numerical calculations. The finalized mesh size ensures there are sufficient nodes at the crack region at all the considered dimensions. Figure 7 shows the comparison of normalized amplitudes of transmitted signals obtained from experimental observations and numerical models for both the inspection frequencies. The arrival time of the leading envelopes and its shape are in good agreement with the experimental observations when the material properties shown in Table 2 were used. The challenging aspect was to determine the exact properties of the PE pipe used in the study because material datasheets usually provide the nominal density value, whereas a wide range of Poisson’s ratio and Young’s modulus for PE material is available in the literature [49]. These values are finalized based on preliminary experiments on the pristine specimen in combination with results from the dispersion analysis of wave modes. The aim was to match the arrival time of the propagating mode in experimental and numerical conditions.

### 4.2. Model Validation

**Figure 8:**
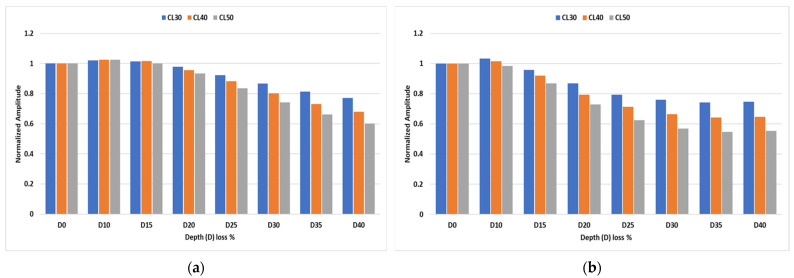
Normalized amplitudes of transmitted signals at (**a**) 35 kHz and (**b**) 50 kHz for different crack depths.

For the amplitude decay analysis, numerical results were generated at different depth losses for all the CLs considered in the experiment. Figure 8 shows the amplitude decay trend of the transmitted signal peaks for the inspection conducted at 35 kHz and 50 kHz, respectively. Similar to experimental observations, a close-to-linear decay trend in the transmitted peak is observed for the 35 kHz signals from 20% depth loss onwards, whereas an initially greater amplitude decay followed by a potential saturation zone at higher crack depths was observed for inspection signals at 50 kHz. 

Table 3 summarizes the amplitude change in terms of the signal peaks at the selected modes compared to the pristine condition from experimental and model results. The results indicate the ability of numerical modeling to capture experimental outcomes with reasonable accuracy for a wide range of crack geometries. A few outliers were observed that could be caused by human errors in data acquisition and the inability to maintain repeated accuracy in achieving precise crack depths in the pipe specimen.

### 4.3. Discussions of Inspection Parameters

It is evident from experimental and numerical results for amplitude decay that the sensitivity of UT towards crack interaction improves as the CL approaches the wavelength of the interacting wave mode, as observed for the CL of 50 mm for both inspection frequencies. The CL of 50 mm represents the case where CL is relatively equal to or slightly higher than the wavelength of the inspecting modes. The decay in the transmitted peak for the 50 kHz signal is relatively higher for all the CDs due to its relatively smaller wavelength compared to the 35 kHz signal. The 50 kHz signal is more sensitive for crack detection at smaller CDs; however, the higher attenuation of the L(0,4) mode may restrict its application for longer-range inspection. For inspection at 35 kHz, wave-crack interaction is not significant until a greater CD is achieved, as observed in the case of the 30 mm CL shown in Figure 5a and Figure 8a. The extent to which crack geometry can limit the sensitivity of UT towards crack interaction can be analyzed by comparing the results of the 30 mm and 50 mm CLs. The maximum change in the amplitude decay with 30 mm CL is up to 40% less compared to that with 50 mm CL. It is recommended that for a wide range of crack geometry inspection, a combination of 35 kHz and 50 kHz signals can be a reasonable choice since a 50 kHz signal offers higher sensitivity at lower CDs whereas 35 kHz offers lower attenuation and a relatively similar level of crack interaction at higher CDs. 

**Figure 9:**
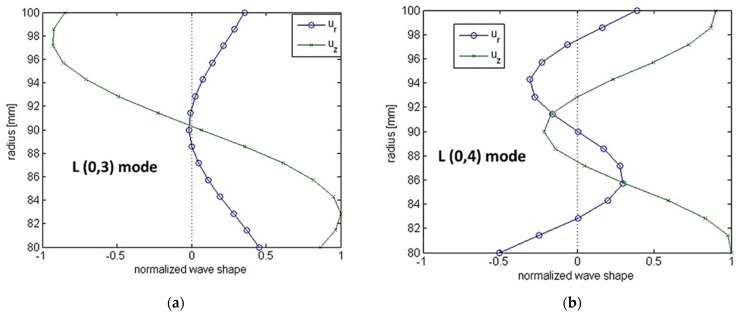
Mode shapes for (**a**) L(0,3) mode at 35 kHz, and (**b**) L(0,4) mode at 50 kHz.

The common trend of linear amplitude decay for the 35 kHz signal with an increasing CD, and a sharper initial amplitude decay with a saturated region at higher CDs for 50 kHz, can potentially be understood by studying the mode shapes of the interacting modes. As shown in Figure 9a, the L(0,3) mode at 35 kHz has a clear linear trend for axial displacement throughout the pipe thickness, hence highlighting the linear decay in the transmitted signal. On the contrary, the L(0,4) mode at 50 kHz has a zone of symmetrical low displacements around the center of the pipe’s thickness, potentially leading to a saturated zone from 30% depth loss onwards, as shown in Figure 9b. It can be projected that the resolution of UT can be limited by CL, especially for the CLs smaller than the wavelength of the interacting mode. The detection of smaller cracks will require smaller wavelength modes; however, the potential of such modes may be restricted by the attenuation challenges and the inherent mode shapes. The displacements along the thickness of pipe might not be suitable for the detection of a wide range of CDs. Therefore, a guided wave physics-based decision needs to be made before finalizing the inspection UT signals.

### 4.4. Effect of Crack Orientation

Numerical simulations can be used to predict experimental outcomes where the fabrication of multiple damage scenarios is not feasible or cost-effective. The signal transmission across the 50 mm CL, 2 mm CW, and 40% depth loss, was used to investigate the effect of crack orientation. The crack orientation was changed in intervals of 15° relative to the longitudinal axis until the crack was oriented perpendicular to the direction of wave propagation. 

**Figure 10:**
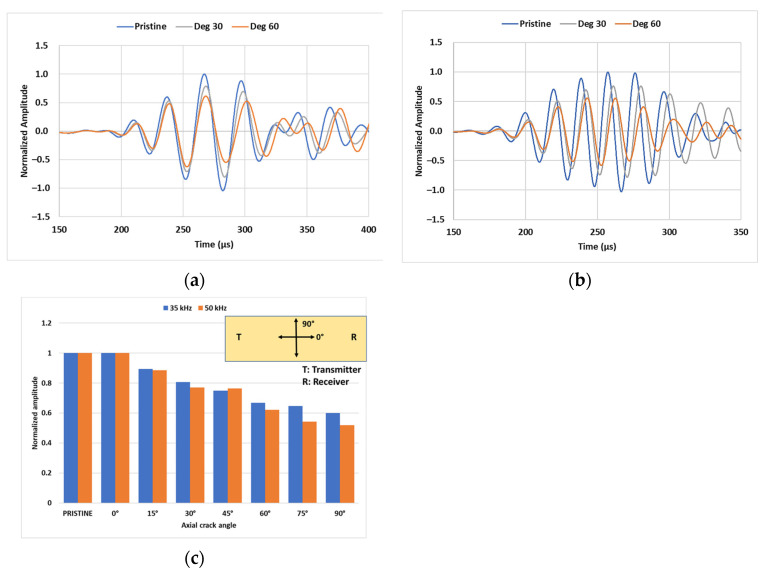
Simulated transmitted signals showing decay for a crack orientation of 30° and 60° at (**a**) 35 kHz inspection and (**b**) 50 kHz inspection; (**c**) amplitude decay trend for different crack orientations from model predictions.

Figure 10a,b show gradual decay with a slight shift in the arrival time in the transmitted amplitude at the crack orientation of 30° and 60° relative to the pristine state of the specimen, for 35 kHz and 50 kHz inspection. This is because of the intrinsic property of longitudinal modes which have dominant displacements in the axial direction; this interaction is the maximum when the crack is perpendicular to the direction of wave propagation. As can be seen in Figure 10c, the decay in the transmitted amplitude reduces by up to 40% when the crack is orientated at 45° compared to its perpendicular state, whereas no change in the transmitted signal was observed for the crack aligned axially (0°). This highlights the potential limitation of exploiting longitudinal modes for crack inspection as axial cracks may go undetected in practice. However, this can potentially be mitigated by using torsional modes instead, which have primary displacements in the circumferential direction and, hence, support the case of wave interaction with the axially aligned crack.

## 5. Conclusions

This study investigated the potential of ultrasonic testing for detecting cracks in PE pipes using laboratory experiments and numerical models. Circumferential cracks with different geometries were fabricated on the external surface of a PE pipe for UT with piezoelectric patches attached on the pipe surface. Finite element models of wave propagation in the PE pipe were developed and calibrated based on experimental observations for further analysis. 

The major findings of the study are as follows:

- The application of UT for monitoring PE pipes can be optimized by the careful selection of inspecting signals using the fundamentals of guided wave physics. The dispersion and attenuation analysis of different types of pipe modes can help isolate the modes with the lowest dispersion and attenuation characteristics. For PE pipelines, L(0,3) and L(0,4) modes are reasonable choices of wave modes for axial pipe inspection.
- The selection of wave modes should be complementary to inspection requirements as the crack geometry plays an important role in the ability of UT. The crack with a length relatively equal to or greater than the wavelength of the inspecting mode is easier to detect by observing the amplitude decay in the transmitted signal; however, a higher crack depth is needed for smaller crack lengths to achieve noticeable decay in the signal amplitude.
- The selection of appropriate PE material properties is critical for developing an accurate simulation model. Laboratory experiments can be performed on pristine specimens to match standard wave mode features, such as group velocity and signal shape, followed by the optimization of the damping properties.
- The wave-crack interaction observed through the decay in peak amplitude is influenced by the orientation of the crack relative to the intrinsic displacements of the wave mode. For longitudinal modes, no wave-crack interaction was observed for axially aligned cracks; hence, the inspection strategies with torsional modes that have displacements in the circumferential direction are desired.

Based on the findings of this study, the potential of UT for PE pipe monitoring is evident. The developed numerical models offer an effective approach to predict experimental outcomes and diversify the application of UT for detecting defects in PE pipes. In the future, the study will be expanded to detect multiple types of damage (internal cracks, multiple cracks) and the damage state of cracks (depth and length) through the automated analysis of transmission signals.

## 6. Remarks and Future Scope of Work

The findings of this study have the potential to establish a foundation for the development of a sophisticated UT setup, enabling cost-effective and time-efficient inspection of natural gas pipelines. This research holds significant importance for pipeline maintenance agencies. One of the current challenges in UT investigations is the lack of specific material properties for PE, which are crucial for developing accurate numerical models. Conventional datasheets for PE typically provide limited information, such as material density, while properties such as the elastic modulus and Poisson’s ratio are unavailable. Additionally, a wide range of properties for the same type of PE can be found in the literature, making it challenging to select the optimal parameters for the developed models. Therefore, the authors recommend conducting preliminary experiments to optimize the material properties, particularly focusing on damping properties that are often overlooked in relevant studies. 

In future research, efforts can be directed towards developing robust models capable of generating synthetic data for experiments in different defect scenarios, as the fabrication time for customized transducers needed for the experiments can be relatively long. Furthermore, the potential of this method can be expanded to detecting cracks of different orientations, different crack locations with respect to the transducers, and internal cracks.

## Figures and Tables

**Table 1:** Input Parameters for GUIGUW software.

Wave Type	Velocity (m/s)	Attenuation Parameters
Longitudinal	2340 (±2%)	0.055 (±5%)
Shear	950 (±5%)	0.29 (±10%)

**Table 2:** Material properties used in the numerical model.

Density (kg/m^3^)	Young’s Modulus (GPa)	Poisson’s Ratio	Rayleigh Damping Parameters
1000	1.9	0.4	α = 15 × 10^3^ at 35 kHz 22 × 10^3^ at 50 kHz; β = 0

**Table 3:** Comparison of transmitted amplitude decay from experimental and model results.

		Amplitude Decay (%)			
Inspection Frequency		35 kHz		50 kHz	
Depth (D) loss %	CL (mm)	Experiment	Simulation	Experiment	Simulation
10	30	−2	2	4	3
	40	−1	2.5	6	2
	50	−3	2	−4	−2
20	30	−6	−2	−13	−13
	40	−5	−4	−11	−20
	50	−14	−6	−28	−27
30	30	−13	−13	−21	−23
	40	−16	−19	−25	−33
	50	−27	−25	−45	−43
40	30	−29	−23	−32	−25
	40	−33	−32	−33	−35
	50	−42	−39	−43	−44

## Data Availability

Data will be available upon reasonable request.
